# The development of a novel bidirectional fine-tuning mandibular advancement device

**DOI:** 10.1186/s12903-024-04619-6

**Published:** 2024-07-26

**Authors:** Huijia Lei, Zijing Wang, Yang Yang, Mo Chen

**Affiliations:** Department of Otorhinolaryngology, Beijing Jishuitan Hospital, Capital Medical University, NO.31 Xinjiekou East Street, Xicheng District, Beijing, 100035 P. R. China

**Keywords:** Mandibular advancement device, Obstructive sleep apnea hypopnea syndrome, Oral orthodontic appliance, Therapeutic outcomes

## Abstract

**Objective:**

To develop a novel mandibular advancement device (MAD) with high comfort, good compliance, and bidirectional fine-tuning capability for patients with obstructive sleep apnea hypopnea syndrome (OSAHS), and to evaluate the therapeutic efficacy of the new MAD.

**Methods:**

The MAD, featuring upper and lower dental splints with a fine-tuning mechanism for mandibular adjustment, incorporates improved design elements such as partial dental coverage, shortened baffles, and memory resin lining. The novel MAD was used to treat 30 OSAHS patients in the study, comparing pre- and post-treatment scores on the Epworth Sleepiness Scale (ESS), the Apnea-Hypopnea Index (AHI), and the lowest oxygen saturation (LSO2).

**Results:**

The novel MAD reduced size and side effects, enhancing comfort. All patients complied well, using it for an average of 95% over 30 days and ≥ 5 h nightly. After treatment, significant improvements were observed in ESS, AHI, and LSO2 (*P* < 0.05).

**Conclusions:**

This novel bidirectional adjustable MAD provides high comfort and compliance, improving treatment precision. It is an effective choice for mild to moderate OSAHS patients and an alternative for those intolerant to CPAP or averse to surgery.

## Background

Obstructive sleep apnea hypopnea syndrome (OSAHS) is a clinically common sleep-related breathing disorder, affecting approximately one quarter of males and one tenth of females globally [1]. Patients with OSAHS experience repeated upper airway obstructions during sleep, leading to decreased blood oxygen saturation [2, 3]. This can induce systemic oxidative stress, inflammatory responses, and enhanced activity of sympathetic nervous system, ultimately resulting in complications in multiple systems such as cardiovascular, cerebrovascular, neurological, endocrine, urogenital, digestive, and hematological systems, severely impacting life quality and even posing a threat to life [4, 5]. Hence, OSAHS has become a critical public health issue during recent years, receiving widespread attention from experts across various disciplines. Currently, continuous positive airway pressure (CPAP) serves as the first choice for OSAHS treatment. Although highly effective, patient tolerance and compliance for CPAP treatment are relatively poor, with 20-50% of patients unable or unwilling to accept CPAP therapy, limiting its long-term clinical effectiveness [6, 7]. The Mandibular advancement device (MAD), a non-invasive treatment method, offers better comfort and compliance compared to CPAP, and is increasingly favored by patients with OSAHS [8–10]. Numerous retrospective studies have shown that, while MAD is less effective than CPAP in reducing disease severity, such as decreasing the Apnea-Hypopnea Index (AHI) and increasing blood oxygen saturation, the mean disease alleviation (MDA) of MAD and CPAP for OSAHS are similar [11–14]. Therefore, MAD has become an effective treatment method for mild to moderate OSAHS, as well as for patients with severe OSAHS who cannot tolerate CPAP and surgical treatments [14, 15].

MADs function by passively fixing the mandible in a forward and downward position, while the contraction of the genioglossus muscle moves the tongue forward, relieving the tongue’s pressure on the soft palate. This ultimately enlarges the pharyngeal airway, thereby achieving the goal of opening and stabilizing the airway. Consequently, with MAD use, the more advancement of the mandible, the greater the improvement should be on the airway opening and therapeutic effect. However, this can lead to discomfort such as temporomandibular joint pain, reducing patient compliance. Conversely, less mandibular advancement increases patient comfort but may decrease treatment effectiveness. Therefore, a persistent challenge in the therapeutic application of MADs for OSAHS has been the optimization of a balance between treatment efficacy and patient comfort. Currently, all MADs domestically and internationally allow only unidirectional forward adjustment from the initial position, which significantly limits the adjustment of patient comfort in clinical applications, affecting long-term efficacy. Furthermore, the most types of MADs are large in size and cause a pronounced foreign body sensation in the mouth, reducing comfort and compliance. Additionally, most MADs cause significant dental pain due to uneven tooth pressure after wearing. Therefore, this study aims to design and develop a novel bidirectional fine-tuning MAD and preliminarily validate its efficacy. By innovating in structure and design, the study seeks to enhance treatment precision, maximize patient comfort, reduce side effects after MAD wearing, improve patient compliance, and ultimately achieve optimal therapeutic outcomes.

## Methods

### The design and production of bidirectional fine-tuning MAD

#### Holistic design

**Fig. 1:**
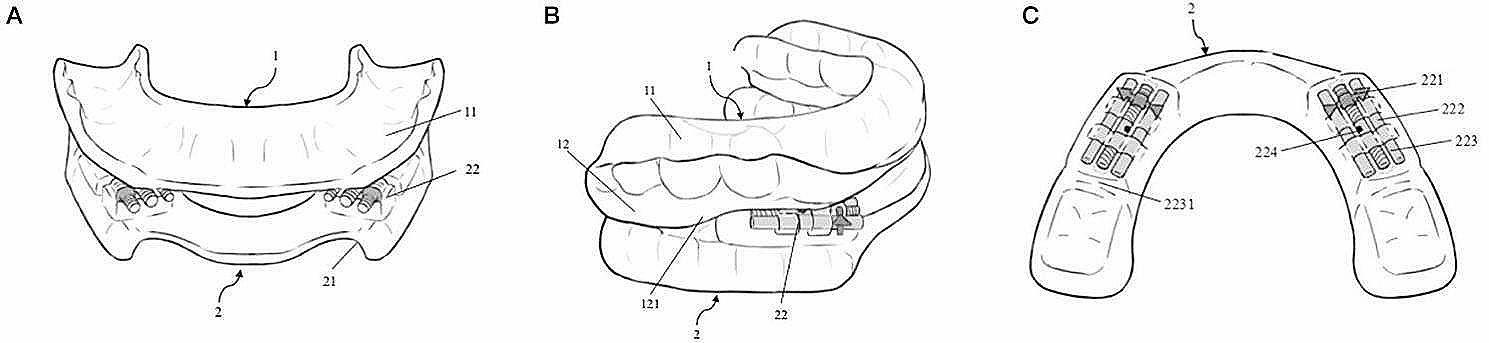
The schematic diagram of (**A**) frontal aspect, (**B**) lateral aspect and (**C**) lower dental splint structure of bidirectional fine-tuning MAD designed in this study. Key to illustrations: 1, upper dental splint; 11, upper alveolar socket; 12, blocking unit; 121, upper inclined surface; 2 lower dental splints; 21, lower alveolar socket; 22, fine-tuning device; 221, fixed part; 222, adjusting part; 223, movable part; 2231, lower inclined surface; 224, adjustment hole

The proposed bidirectional fine-tuning MAD in this study consists of upper and lower dental splints. The design of these splints is a non-full dental arch coverage type, covering only four teeth on each side of the upper and lower jaws (the 3rd to 6th teeth), resulting in each splint having four dental sockets on both the left and right sides. The upper splint is equipped with a downward-protruding blocking unit, the width of which is the same or similar to that of the upper splint and includes an upper inclined surface. The lower splint features an upward-protruding adjustment unit, the width of which is the same or similar to that of the lower splint and contains a fine-tuning device and a lower inclined surface. The upper inclined surface of the blocking unit and the lower inclined surface of the adjustment unit interact to allow the lower splint to occlude with the upper splint, enabling relative movement between the upper and lower trays (Fig. 1A and B).

#### Fine-tuning device design

The fine-tuning device is situated on the occlusal surface between the third and fourth anterior teeth of the lower dental splint. The device comprises a fixed part, an adjusting part, and a movable part. The adjusting part consists of an archwire screw, and the movable part is flexibly connected to the fixed part via the adjusting part. The initial state between the fixed and movable parts of the fine-tuning device is set to a separation of 1–2 mm. One end of the adjusting part is embedded in the fixed part, while the other end is embedded in the movable part, facilitating bidirectional adjustment. Orthocryl, the orthodontic base polymer, is applied in layers to cover and complete the molding of the upper and lower dental splints, with the fine-tuning device being embedded within the lower splint (Fig. 1C). Finally, a layer IMPAK (Nobilium, USA), the tooth-protective memory resin is lined on the inner surfaces of the upper and lower dental splints. IMPAK is mixed with a corresponding liquid in a specific ratio and, when in a viscous liquid state, is poured into the splint. It is then fixed and occluded using a dental impression, ultimately resulting in the final molding.

### Preliminary efficacy validation of bidirectional fine-tuning MAD

#### Study population

In accordance with ethical standards, the patients diagnosed with OSAHS who exhibit snoring were selected for assessment of blockage level. The study focused on patients primarily experiencing oropharyngeal obstruction level. Inclusion criteria were as follows: (1) 18–70 years; (2) Resistance or intolerance to CPAP therapy or surgical treatment; (3) Apnea-Hypopnea Index (AHI) ≥ 5; (4) Predominant airway obstruction at the oropharyngeal level; (5) Ability to communicate effectively and provide informed consent. Exclusion criteria included: (1) Severe periodontal disease or temporomandibular joint disorders; (2) Tooth loosening greater than Grade II, or fewer than 10 teeth in one jaw, rendering oral orthotics unsuitable; (3) Unstable systemic diseases such as chronic obstructive pulmonary disease, severe cardiac arrhythmia, autoimmune disorders, tumors in the peri-upper airway area; (4) Psychiatric disorders or poor compliance; (5) Grade III tonsillar hypertrophy. Ultimately, 30 OSAHS patients were enrolled, including 10 with mild severity (5 ≤ AHI ≤ 15), 10 with moderate severity (15 < AHI ≤ 30), and 10 with severe severity (AHI > 30). In our study, oropharyngeal obstruction as a selection criterion was determined through a comprehensive evaluation process. This included taking detailed patient histories, conducting thorough physical examinations using the Mallampati classification, performing fiberoptic nasopharyngoscopy to visualize the airway, utilizing polysomnography (PSG) to confirm obstructive events. This multi-faceted approach ensured accurate identification of oropharyngeal obstruction among study participants, enhancing the reliability of our findings. The study protocol was reviewed and approved by the Ethics Review Committee of Beijing Jishuitan Hospital (approval number: K2023-364) and adhered to the principles of the Declaration of Helsinki.

#### Evaluating criteria of efficacy

All enrolled patients with OSAHS completed the Epworth Sleepiness Scale (ESS) prior to treatment to establish baseline sleepiness scores. The ESS, which assesses the level of daytime sleepiness, consists of 8 items scored from 0 to 3, yielding a total score range of 0–24; higher scores indicate more severe sleepiness. Customization of bidirectional micro-adjustment MAD were provided for all patients. Following MAD titration and a one-month adaptation period, a second polysomnography (PSG) was conducted to record changes in AHI and the lowest blood oxygen saturation (LSaO2), along with weekly device usage duration. Efficacy was determined by the degree of improvement in AHI according to the ‘2009 Guidelines for the Diagnosis and Surgical Treatment of Obstructive Sleep Apnea Hypopnea Syndrome’ [16, 17]: Cure is defined as AHI < 5 times/hour; Effective is defined as AHI < 20 times/hour with a reduction of ≥ 50%. The total efficacy rate is calculated as (Cured + Effective cases) / Total number of patients × 100%. Cure rate = (Number of cured patients) / Total number of patients × 100%.

In our study, the second PSGs were conducted as full overnight studies, adhering to the guidelines established by the American Academy of Sleep Medicine (AASM). These comprehensive studies included continuous monitoring of various physiological parameters such as airflow, respiratory effort, oxygen saturation, electroencephalography (EEG), electrooculography (EOG), electromyography (EMG), and electrocardiography (ECG). This approach ensured a thorough evaluation of sleep architecture and respiratory events, providing accurate and reliable data for the assessment of OSAHS. By following the AASM guidelines, we ensured the highest standard of sleep study methodology, enhancing the validity and reproducibility of our findings.

### Statistical analysis

Statistical analysis was performed using SPSS version 24.0. The data that followed a normal distribution were presented as mean ± standard deviations, while skewed and categorical data were described using medians and interquartile ranges. Comparisons of treatment efficacy across different severity levels were conducted using Chi-square tests or Fisher’s exact test.

## Results

### Demographic and anthropometric characteristics

The detailed information was presented in Table 1. The study included a total of 30 participants, divided into three groups based on the severity of their condition: mild (*n* = 10), moderate (*n* = 10), and severe (*n* = 10). The mean age of participants was 47.0 ± 14.2 years in the mild group, 50.4 ± 10.6 years in the moderate group, and 44.9 ± 13.1 years in the severe group, with no significant difference between groups (*P* = 0.627). Gender distribution showed a trend towards a higher proportion of males in the severe group (90.0%) compared to the mild (40.0%) and moderate (60.0%) groups, although this difference did not reach statistical significance (*P* = 0.089).

**Table 1:** Demographic, anthropometric, and clinical characteristics of patients with different severities of OSAHS. BMI, body mass index; AHI, apnea-hypopnea index

Indices	Mild (*n* = 10)	Moderate (*n* = 10)	Severe (*n* = 10)	*P*
Age	47.0 ± 14.2	50.4 ± 10.6	44.9 ± 13.1	0.627
Gender				0.089
Male	4 (40.0)	6 (60.0)	9 (90.0)	
Female	6 (60.0)	4 (40.0)	1 (10.0)	
BMI	24.4 ± 2.0	25.8 ± 2.0	29.8 ± 4.7	0.002
Height (m)	1.6 ± 0.1	1.7 ± 0.1	1.70.1	0.175
Body weight (kg)	66.7 ± 10.6	75.2 ± 9.6	88.1 ± 19.1	0.006
AHI reduction rate (%)	67.9 ± 12.7	59.4 ± 21.2	58.3 ± 24.9	0.519
Efficacy [n(%)]				< 0.001
Effective	0	6 (60.0)	4 (40.0)	
Cure	10 (100.0)	3 (30.0)	1 (10.0)	
Ineffective	0	1 (10.0)	5 (50.0)	
Wearing time (d)	28.9 ± 1.0	27.9 ± 1.0	28.5 ± 1.2	0.124
Daily usage time (h)	6.2 ± 0.8	5.5 ± 0.8	6.2 ± 1.0	0.151

Body Mass Index (BMI) differed significantly across the groups, with values of 24.4 ± 2.0 kg/m² for the mild group, 25.8 ± 2.0 kg/m² for the moderate group, and 29.8 ± 4.7 kg/m² for the severe group (*P* = 0.002). Height was similar among the groups, with means of 1.6 ± 0.1 m for the mild group, 1.7 ± 0.1 m for the moderate group, and 1.7 ± 0.1 m for the severe group (*P* = 0.175). Body weight also showed significant variation, with mean values of 66.7 ± 10.6 kg for the mild group, 75.2 ± 9.6 kg for the moderate group, and 88.1 ± 19.1 kg for the severe group (*P* = 0.006).

The Apnea-Hypopnea Index (AHI) reduction rate did not differ significantly between groups, with rates of 67.9 ± 12.7% for the mild group, 59.4 ± 21.2% for the moderate group, and 58.3 ± 24.9% for the severe group (*P* = 0.519). However, the efficacy of the treatment, categorized as effective, cure, or ineffective, showed significant differences (*P* < 0.001). In the mild group, all participants (100.0%) were cured. In the moderate group, 6 participants (60.0%) were classified as effective, 3 (30.0%) as cured, and 1 (10.0%) as ineffective. In the severe group, 4 participants (40.0%) were effective, 1 (10.0%) was cured, and 5 (50.0%) were ineffective.

The wearing time was consistent across the groups, with a mean of 28.9 ± 1.0 days for the mild group, 27.9 ± 1.0 days for the moderate group, and 28.5 ± 1.2 days for the severe group (*P* = 0.124). Daily usage time also did not significantly differ, averaging 6.2 ± 0.8 h for the mild group, 5.5 ± 0.8 h for the moderate group, and 6.2 ± 1.0 h for the severe group (*P* = 0.151).

### Development and evaluation of a novel bidirectional fine-tuning MAD for OSAHS

**Fig. 2:**
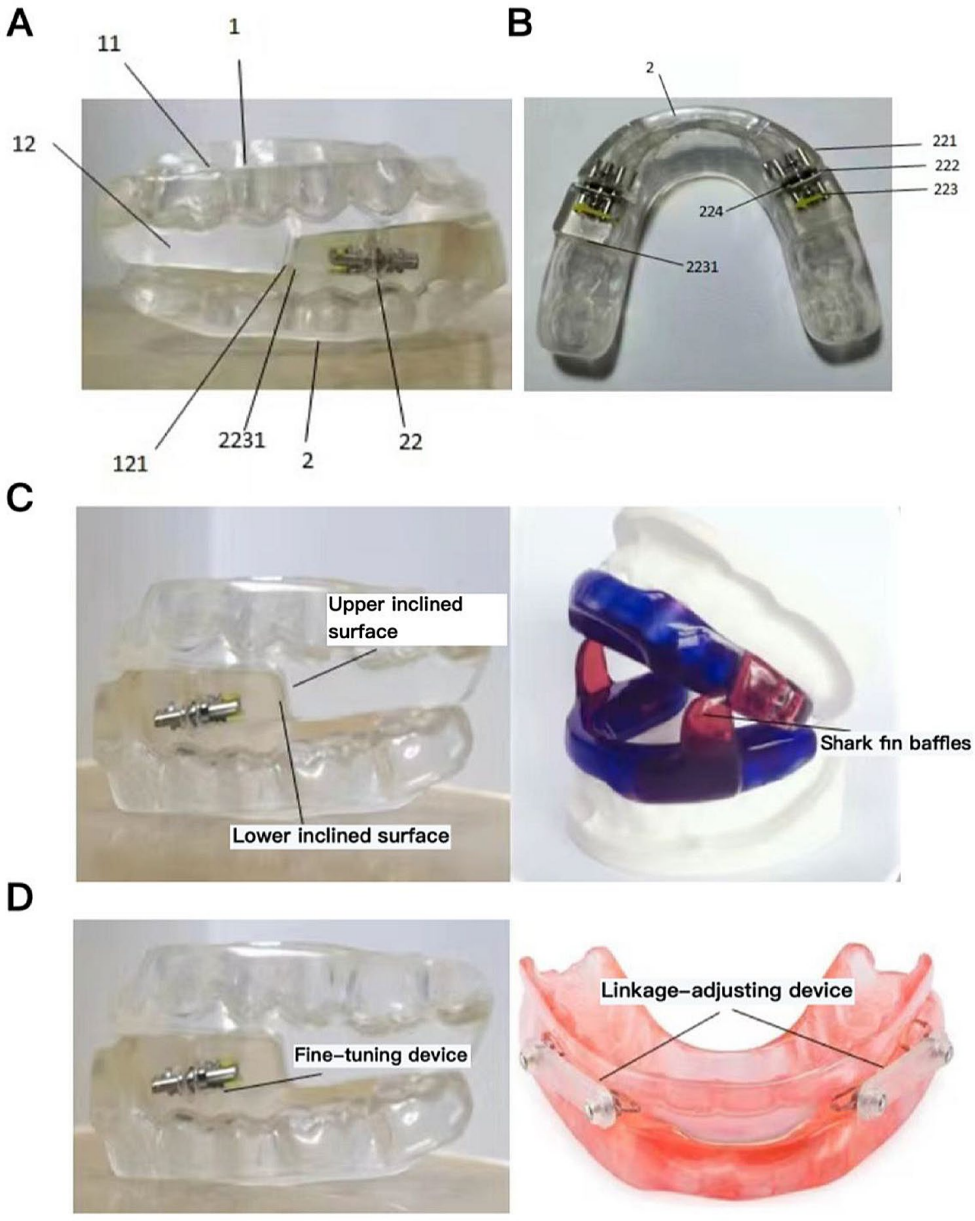
The actual image of the (**A**) lateral aspect, (**B**) lower dental splint in the bidirectional micro-adjustable MAD designed in this study. (**C**) Comparative diagram of the bidirectional fine-tuning MAD with shark-style MAD. (**D**) Comparative diagram of the bidirectional fine-tuning MAD with linkage-adjusting MAD. Key to illustrations: 1, upper dental splint; 11, upper alveolar socket; 12, blocking unit; 121, upper inclined surface; 2 lower dental splints; 21, lower alveolar socket; 22, fine-tuning device; 221, fixed part; 222, adjusting part; 223, movable part; 2231, lower inclined surface; 224, adjustment hole

In this study, a novel bidirectional fine-tuning MAD was successfully developed (Fig. 2A and B). On one hand, bidirectional adjustment was achieved by setting a 1–2 mm initial gap between the fixed and movable parts of the micro-adjustment mechanism. This allows the mandible to move forwards or backwards from its initial protruded position after the patient wears the MAD. Furthermore, the design of the fine-tuning device, positioned between the biting surfaces of the third and fourth anterior teeth on the lower dental splints, not only reduces the volume of the MAD, but also avoids the need to elevate the occlusal relationship, thereby reducing pressure on the temporomandibular joint. On the other hand, by modifying the shark-like fin baffle into inclined upper and lower surfaces, mandibular movement relative to the maxilla is facilitated, further reducing the size of the device, lowering the sensation of foreign bodies in the mouth, and enhancing patient comfort. This design also accommodates patients with smaller oral cavities (Fig. 2C). Additionally, the upper and lower dental splints are designed as non-full dental arch coverings, preventing discomfort such as tooth pain due to uneven force distribution caused by irregular anterior dental arches. The inner surfaces of the splints are lined with a layer of IMPAK, an elastic resin that protects the teeth, increases softness, and buffers the pressure exerted by the MAD, thereby further enhancing comfort. Finally, the newly designed bidirectional MAD in this study allows for more precise treatment. The commonly used inkage-adjusting device is replaced with a fine-tuning device, which can be adjusted at the millimeter level. Each adjustment advances the mandible by 0.25 mm, significantly increasing the precision of the treatment (Fig. 2D).

In this study, 30 patients demonstrated good compliance with the novel bidirectional fine-tuning MAD, with an average usage of 95% of the days within a 30-day period and ≥ 5 h per night, without any adverse events. Among these 30 patients, discomfort symptoms while wearing the MAD included minor tooth pain (1/30), which generally resolved within a few hours after removing the MAD; drooling (3/30), which subsided within a week; and temporomandibular joint soreness (4/30), which disappeared two days after adjusting the forward movement of the MAD by bidirectional fine-tuning mechanism. Throughout the treatment period with the MAD, no patients experienced tooth loosening, and the MAD remained well-fixed without loosening or slipping.

The therapeutic outcomes for the three groups of patients wearing the MAD are shown in Table 2. For the mild OSAHS group, the cure rate was 100% (10/10), and the total effective rate was 100% (10/10); for the moderate OSAHS group, the cure rate was 30% (3/10), and the total effective rate was 90% (9/10); for the severe OSAHS group, the cure rate was 10% (1/10), and the total effective rate was 50% (5/10). Comparative data before and after wearing the MAD for the three groups (Tables 3 and 4) showed that the quartile values of AHI were significantly reduced after treatment in all groups, with statistical significance (mild group, Z=-2.807, *P* = 0.005; moderate group, Z=-2.803, *P* = 0.005; severe group, Z=-2.803, *P* = 0.005). The quartile values of LSO2 significantly increased after treatment (mild group, Z=-2.812, *P* = 0.005; moderate group, Z=-2.812, *P* = 0.005; severe group, Z=-2.499, *P* = 0.012). The quartile values of ESS significantly decreased after treatment (mild group, Z=-2.812, *P* = 0.005; moderate group, Z=-2.214, *P* = 0.027; severe group, Z=-2.809, *P* = 0.005).

**Table 2:** Comparison of therapeutic efficacy in patients with varying degrees of OSAHS Severity

	Ineffective	Effective	Cure	Overall efficacy rate(%)	Cure rate(%)	*P* value
Mild	0	10	10	100	100	< 0.001
Moderate	1	9	3	90	30	< 0.001
Severe	5	5	1	50	10	< 0.001
Total	6	24	14	80	46.67	< 0.001

**Table 3:** Comparison of various indicators before and after MAD Treatment in patients with different OSAHS severity. AHI, apnea-hypopnea index; LSO2, lowest oxygen saturation; ESS, Epworth sleepiness scale

Indices	Mild			Moderate			Severe		
	Percentiles			Percentiles			Percentiles		
	p25	p50	p75	p25	p50	p75	p25	p50	p75
AHI									
Before	5.9	10.8	14.2	16.8	25.8	27.3	38.5	64	79.5
After	2.8	3.5	3.9	4	9	12.8	10.2	18.3	30.4
% Change	-52.5	-67.6%	-72.5	-76.2%	-65.1%	-53.1%	-73.5%	-71.4%	-61.8%
LSO2 (%)									
Before	81	82	87	73.5	81	84.3	57.5	66	81
After	86	89.5	92	83.5	86	90	69.25	81.5	87
% Change	+ 6.2%	+ 9.2%	+ 5.6%	+ 13.6%	+ 6.2%	+ 6.8%	+ 20.4%	+ 23.5%	+ 7.4%
ESS									
Before	6	9	14.3	10.8	15.5	18.8	16.8	19	20.3
After	1	2	4	3.5	10	14	6.8	9.5	13
% Change	-83.3%	-77.8%	-72.0%	-67.6%	-35.5%	-25.5%	-59.5%	-50.0%	-36.0%

**Table 4:** Statistical metrics and p value of various indicators in patients with different OSAHS severity. AHI, apnea-hypopnea index; LSO2, lowest oxygen saturation; ESS, Epworth sleepiness scale

Diagnostic grading		LSO2	ESS	AHI
Mild	Z	-2.812	-2.812	-2.807
	p	0.005	0.005	0.005
Moderate	Z	-2.812	-2.214	-2.803
	p	0.005	0.027	0.005
Severe	Z	-2.499	-2.809	-2.803
	p	0.012	0.005	0.005

## Discussion

Currently, there are many types of MADs used in clinical practice, with a variety of classification methods. The treatment efficacy varies based on the design of the MADs [18]. According to the customization method, MADs can be divided into custom-made MADs and prefabricated MADs. Custom-made MADs are individually tailored using impression materials or scans of the upper and lower dental arches to gather patient-specific oral information. Prefabricated MADs, similar to boil-and-bite or thermoplastic orthotics, do not require fabrication and can be self-fitted by the patient. Studies have shown that custom-made MADs are more effective in improving the AHI compared to prefabricated thermoplastic MADs, and they have significantly reduced side effects during the initial weeks of wearing [19]. Based on the structure of MADs, they can be classified into monobloc and bi-bloc types. Monobloc MADs have a fixed degree of mandibular advancement; after the upper and lower teeth are positioned, the mandible is fixed in a downward and forward position, without the ability to adjust movement in other directions. If discomfort occurs after wearing, the device cannot be modified and must be remade. Bi-bloc MADs consist of two separate upper and lower parts that contact each other after positioning, allowing the mandible to move forward within a certain range. Depending on whether the mandibular position can be adjusted after MAD placement, they are further divided into adjustable and non-adjustable MADs. Most MADs used today are custom-made, bi-bloc, and adjustable, incorporating linkage or fine-tuning devices. These features allow for self-regulation of mandibular advancement to enhance comfort, improve therapeutic outcomes, and reduce adverse reactions [19–21].

However, current MADs still exhibit limitations and side effects in their usage, including the following aspects. Firstly, all MADs, both domestically and internationally, can only be adjusted unidirectionally from the initial position, moving the mandible forward, without the capability to move it backward from the initial position. This significantly restricts the adjustment of patient comfort. During the fabrication of MADs, there is no uniform standard for determining the initial mandibular protrusion amount. It is often based on an empirical occlusal experiment, where patients are instructed to protrude their mandible to the extreme position. The measured data is recorded as the maximum protrusion amount. Subsequently, patients are instructed to gradually retract their mandible until reaching a specific comfortable occlusal position, maintaining it for several minutes. Through patient self-feedback, the most comfortable initial protrusion amount is obtained. Most scholars believe the initial position of MAD to be 50–75% of the maximum protrusion amount [22–24]. Then the MAD is fabricated based on the initial protrusion amount acquired from the occlusal experiment. Therefore, after the MAD is fitted, the patient’s mandible is in a certain protruded state, which is gradually adjusted forward to achieve the maximum therapeutic effect within the physiological tolerance. Since patients need to wear the MAD for several hours during nighttime sleep, the most comfortable initial protrusion amount during a few minutes of the occlusal experiment may not be the same for several hours. Thus, some patients require the mandible to be adjusted backward from the initial position after wearing the MAD. Currently, there are no bidirectionally adjustable MADs available domestically or internationally. Secondly, the internationally recognized shark-type MADs, due to the presence of the shark-like fin baffle, result in a larger overall size of the orthodontic appliance, which causes significant foreign body sensation in the mouth for Asians with smaller oral cavities. Thirdly, all current MAD designs involve full dental arch coverage, which causes significant tooth pain and discomfort due to uneven pressure, especially in patients with misaligned anterior teeth. Therefore, exploring a new type of adjustable, safe, and effective MAD, continuously improving patient comfort and compliance, has great clinical significance in enhancing the treatment efficacy of OSAHS.

This study addresses the existing issues in the clinical application of MADs by designing and fabricating a novel bidirectional fine-tuning MAD. The design structure is innovatively improved to maximize patient comfort, reduce side effects, and enhance patient compliance. Previous literature reported a compliance rate of 83% for semi-customized non-adjustable MADs in treating OSAHS with an average usage of 4.4 h/d, whereas personalized adjustable MADs showed a compliance rate of 92% with an average usage of 5.7 h/d [25]. In this study, 30 OSAHS patients were instructed to wear the MAD for one month, resulting in a compliance rate of 95% and an average nightly usage of ≥ 5 h. This may be attributed to the high comfort of the bidirectional fine-tuning MAD, minimal side effects, absence of severe adverse reactions, and the patients’ ability to autonomously adjust the mandibular advancement. The study preliminarily validated the clinical efficacy in 30 OSAHS patients. Both mild and moderate patients achieved good therapeutic effects with an efficacy rate exceeding 90%, likely due to the high precision, comfort, and compliance associated with the bidirectional fine-tuning MAD. The overall efficacy rate for severe patients was 50%, possibly related the obstruction of multiple levels commonly present in severe cases, as MADs are more suitable for patients with oropharyngeal obstruction. Additionally, after MAD treatment, both mild to moderate and severe patients showed significant improvement in daytime sleepiness symptoms, with a notable decrease in the ESS scores, and in sleep monitoring indicators, including a substantial reduction in the AHI and a notable increase in the LSO2. In summary, this study is the first domestically and internationally to fabricate a bidirectional fine-tuning MAD. Through design improvements, the bidirectional adjustment of MAD significantly enhanced comfort, fostering patient adherence and yielding favorable therapeutic outcomes.

Despite significant achievements in designing and fabricating the bidirectional fine-tuning MAD, this study has several limitations. Firstly, the sample size is small, with only 30 OSAHS patients, limiting the generalizability of the results. Larger-scale studies are necessary to validate the effectiveness and safety of the MAD. Secondly, the follow-up period is short, only one month, which is insufficient to assess long-term effects and potential side effects. Extended follow-up is needed for a comprehensive evaluation. Additionally, the study lacks direct comparative studies with other existing MADs. While the bidirectional fine-tuning MAD shows superior comfort and compliance, its relative advantages need further verification. Future research should include comparative trials to clarify its unique benefits and applicability. Moreover, the method for determining the initial mandibular protrusion amount relies on empirical occlusal experiments, lacking standardization. This could lead to variability in comfort and efficacy. Future studies should develop more objective and standardized methods to optimize initial protrusion, enhancing comfort and outcomes. Finally, the study did not fully consider patient heterogeneity. Variations in oral structure, dental alignment, and mandibular movement range can affect patient responses. Future research should analyze responses from different patient groups to provide personalized treatment plans.

## Data Availability

The data used to support the findings of this study are included within the article.
